# Surgical Treatment by Partial Petrosectomy for a Middle-Ear Carcinoid with Progressive Extension: A Case Report and Review of the Literature

**DOI:** 10.1155/2010/818673

**Published:** 2010-06-06

**Authors:** Mitsuhiro Aoki, Keisuke Mizuta, Natsuko Ueda, Nansei Yamada, Yatsuji Ito, Hiroki Kato, Yoshinobu Hirose

**Affiliations:** ^1^Department of Otolaryngology, Gifu University Graduate School of Medicine, 1-1 Yanagido, Gifu City 501-1194, Japan; ^2^Department of Radiology, Gifu University Graduate School of Medicine, 1-1 Yanagido, Gifu City 501-1194, Japan; ^3^Department of Tumor Pathology, Gifu University Graduate School of Medicine, 1-1 Yanagido, Gifu City 501-1194, Japan

## Abstract

We herein report a 59-year-old male patient with a recurrent carcinoid tumor of the middle ear 7 years after a tympanomastoidectomy. The CT and dynamic MRI demonstrated an extensive tumor close to the carotid artery canal and the jugular bulb, and the tumor was removed by a partial petrosectomy with a transmastoid approach. The histopathological findings revealed a solid and trabecular tumor with cells positive for cytokeratin, chromogranin A, synaptophysin, and CD56. The MIB-1 antibody for the Ki-67 antigen was positive in 6.6% of the tumor cells. The relevant literature is reviewed in regard to the present case.

## 1. Introduction

Carcinoid tumors primarily originate from gastrointestinal organs and the trachea. Carcinoid tumors of the middle ear are very rare; however, there have been more than 50 reports of such tumors since 1980 [1]. A middle-ear carcinoid tumor is usually confined to the tympanum, and osteolytic extension of the tumor is rare [2]. Several patients show osteolytic invasion and cervical lymph node metastasis, suggesting that the middle-ear carcinoid should be classified as a low-grade malignancy [3–5]. The current report presents a patient with extensive osteolytic enlargement of a middle-ear carcinoid close to the jugular bulb and carotid artery canal, and also reviews the previous studies of carcinoid tumors of the middle ear. 

## 2. Case Report

**Figure 1:**
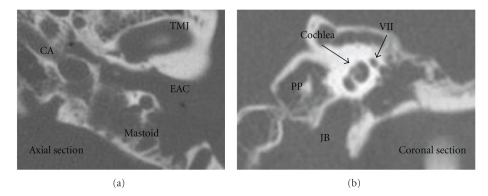
CT. The tympanum and mastoid were filled with an isodensity shadow with bone erosion. The wall of the carotid artery and jugular bulb appeared to be thick and erosive. CA: carotid artery, JB: jugular bulb, TMJ: temporomandibular joint, EAC: external auditory canal, VII: the seventh nerve, PP: Petrous pyramid.

**Figure 2:**
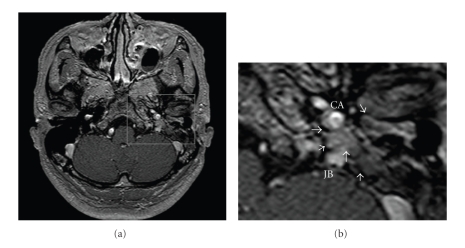
Dynamic enhanced MRI. The mass close to the carotid artery and jugular bulb through the tympanum and mastoid was enhanced in the early phase of the dynamic MRI (white arrows).

A 59-year-old male patient presented with ear pain and bleeding of the left ear, and upon closer investigation a reddish bulging mass extending through the left tympanic membrane from the middle ear was observed. The pure tone audiogram showed an 80-dB mixed hearing loss with an increased threshold of bone conduction in the high tone frequency range. The patient experienced no dizziness or facial palsy. The tympanum and mastoid were filled with an isodensity shadow indicating bone erosion, and the wall of the carotid artery canal and the jugular bulb appeared to be thick and erosive on CT (Figure 1). The mass was close to the carotid artery and jugular bulb through the tympanum, and the mastoid space was enhanced in the early and late phases of the dynamic MRI. The enhanced mass also appeared on the underside of the promontory of the middle ear (Figure 2). The patient had experienced a tympanomastoidectomy for tumors in the tympanum 7 years previously and the pathological diagnosis was adenoma of the middle ear.

The surgical findings revealed that a grayish-red tumor with a slight yellowish hue filled the mastoid. The upper construction of the stapes was conservative, although it was covered with granulation. We performed a canal wall-down mastoidectomy to expose the sigmoid sinus, which revealed the tumor mass close to the jugular bulb. The tumor had originated from the mucous membrane of the hypotympanum and progressed to destroy the bony portions of the posterior wall of the extra meatus through the underside of the cochlear promontory with communication between the hypotympanum and mastoid. There was bone erosion in the tympanic portion of the facial nerve canal, but no invasion to the facial nerve and jugular bulb was observed. Removal of the bony annulus and the residual tumors in the hypotympanum revealed the internal carotid artery with bony erosion, and the tumor was completely removed, sparing facial nerve. 

**Figure 3:**
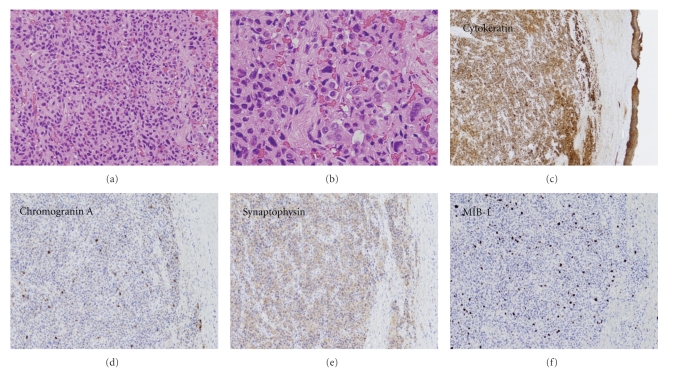
Pathological findings. The histopathological findings revealed a solid tubuloglandular pattern, resembling an adenomatous tumor of the middle ear ((a) examination on low power). One cell type, the A-type cells lining the glandular lumina, was observed with a lower frequency. These slender darkly staining cells had the appearance of endothelial cells. The other cells, the B-type cells, were observed in glandular structures and were characterized by a round or oval nucleus and an abundant, pale cytoplasm ((b) examination on high power). The B-type cells were typically positive for cytokeratin, chromogranin A, and synaptophysin, and 6.6% of the carcinoid tumor cells were positive for MIB-1 staining.

The histopathological findings showed a solid sheet of homogenous cells, which was surrounded by a fibrous border. The tumor cells had round, oval, or slightly irregular nuclei with finely-dispersed chromatin, and occasionally formed glandular or tubular structures (Figures 3(a) and 3(b)). They were typically positive for cytokeratin, chromogranin A, synaptophysin, and CD56, but were negative for S-100. The proliferative capacity of the tumor cells was assessed by observing the cells expressing the marker MIB-1, which is an antibody against antigen Ki-67. This was used to calculate the proliferation index for each tumor lesion by counting the total number of tumor cell nuclear profiles and the number of MIB-1-positive nuclear profiles in randomly and systematically selected fields. The first field in each tumor lesion was selected randomly, and the following fields were sampled systematically using a mesh [6]. The positive rate of MIB-1 was 6.6% (Figure 3). The tumor was diagnosed as carcinoid tumor based on these pathological findings.

## 3. Discussions

Murphy described the first case of a carcinoid tumor of the middle ear in 1980 [1], and approximately 50 cases of middle-ear carcinoid have been reported since. More than 90% of the patients complain of hearing loss, and 20%–30% of patients suffer from ear fullness, tinnitus, and ear discharge. They usually present with conductive hearing loss, whereas a few subjects had sensorineural hearing loss, as appeared in the present patient. Transient facial nerve palsy is rarely observed. A carcinoid tumor generally develops slowly, and the average disease duration is as long as 28 months [7]. 

A carcinoid tumor shows an isodensity shadow equivalent to the otitis media and cholesteatoma on CT. A carcinoid rarely shows osteolytic local-regional spread and destruction of the ossicles, which are helpful for differentiating the carcinoid from adenocarcinomas and cholesteatomas. However, patients with extensive osteolytic spread of a middle-ear carcinoid as in the current subject have been reported [3, 4, 8, 9]. The MRI findings of the carcinoid tumors may not be useful for differentiation from other inflammatory diseases and other tumors; however, dynamic enhanced MRI in the present case showed carcinoid tumors to be enhanced earlier after the administration of Gd than granulation and other types of normal tissue. In addition, dynamic enhanced MRI has been reported to be useful for the differentiation of tumor recurrence and posttreatment fibrosis in regions other than the head and neck [10]. Recent studies demonstrated that dynamic enhanced MRI is valuable for the differentiation of posttreatment fibrosis and tumor recurrence in the head and neck regions, as in other regions [11, 12]. Therefore, our case report also supports the finding that dynamic enhanced MRI is helpful for detecting the local extension of middle-ear carcinoids and is valuable for differentiation of tumor recurrence and posttreatment fibrosis. Nuclear modalities such as scintigraphy with octreotide and PET scanning are also clinically efficient for detecting the recurrence and metastasis of carcinoid tumors [13, 14].

The pathological diagnosis of carcinoid tumors is based on light microscopy and is confirmed by immunohistochemical evaluation for differential diagnoses including adenoma, paraganglioma, and adenocarcinoma. However, it is not always easy to distinguish between middle-ear carcinoids and middle-ear adenomas. One report noted that it is therefore reasonable to regard both tumors as two faces of a single tumor type [15]. The histology of middle-ear carcinoids is similar to other carcinoids, and shows a solid sheet of monotonous cells surrounded by a fibrous border. The tumor cells, which have round, oval, or slightly irregular nuclei with finely-dispersed chromatin occasionally form glandular or tubular structures. They are strongly positive for cytokeratin, which is an epithelial tumor marker, and for neuroendocrinological markers such as chromogranin A, synaptophysin, CD56, and vimentin, but are negative for S-100. These tendencies are helpful for differentiating such tumors from paraganglioma, because paraganglioma is usually positive for S-100 [2, 16]. 

Most of the typical carcinoid tumors do not show a proliferative index detected by Ki-67 labeling using MIB-1 indices exceeding 10%, and most small-cell carcinomas show substantially higher values than 25% [17–19]. The MIB-1 positive rate in the current case was 6.6%, thus resulting in a diagnosis of a typical carcinoid. 

The complete removal by a tympanomastoidectomy or radical mastoidectomy is considered the optimal treatment for middle-ear carcinoid tumors. However, there is no established approach to therapy for middle-ear carcinoids because of the small number of cases. A conservative local resection is reported to be sufficient for patients with small-bowel carcinoid tumors (less than 2 cm) [20]. The middle-ear carcinoid tumors are generally circumscribed; therefore, the basic principles in treating middle-ear carcinoid tumors may be similar to the treatments for small carcinoid tumors of other organs. 

Although local invasion of the middle-ear carcinoid is usually slow and nondestructive, 4 reported patients underwent surgical treatments by petrosectomy for the invaded tumors with extensive enlargement. One patient was initially treated with a petromastoidectomy for a tumor in the hypotympanicum extending into the external auditory canal without bone destruction [8], and another patient initially underwent a partial petrosectomy for the removal of middle-ear tumors extending into the hypotympanicum with partial erosion of the stapes superstructure [9]. Knerer et al. described surgical treatment by extended subtotal petrosectomy for an extensive recurrent tumor with a close relationship to the tegmen tympani, facial nerve, and the ascending and horizontal portions of the carotid canal [3]. Menezes et al. performed a craniotomy, petrosectomy, neck dissection, and parotidectomy for a recurrent carcinoid tumor, but the carcinoid tumor recurred within 3 months after the surgery [4]. In the present case, the carcinoid tumor was close to the carotid artery and jugular bulb through its large extension involving the residual hypotympanic peritubal and perilabyrinthine cells. Radical resection of the carcinoid tumor was possible by a partial petrosectomy, furthermore sparing the facial nerve. 

To date, adjuvant radiotherapy was administered in aggressive cases with middle-ear carcinoid tumors, but the clinical efficacy of this treatment modality has not yet been established [3–5, 7]. Our patient therefore underwent no adjuvant radiotherapy.

Six of 34 reported patients that underwent surgical treatment had a recurrence of the carcinoid tumor of the middle ear, and the average length of time between the initial treatment and the reappearance of the recurrent lesion was 200 months. The length of time between the initial treatment with a tympanomastoidectomy or radical mastoidectomy and the recurrence was relatively long (approximately 15–33 years) [7]. These results suggest that an extended follow-up period is necessary, even in patients where a nearly complete resection was performed, since carcinoid tumors of middle ear slowly increase in size. 

Regional metastatic disease occurred several years after the initial treatment in 5 patients, including 2 patients with metastases to the intraparotid gland lymph nodes, and 3 patients with metastases to the cervical lymph nodes. All cases were surgically managed with a parotidectomy or a neck dissection [4, 5, 7, 21]. However, one subject with an intraparotid gland lymph node metastasis underwent a craniotomy for the extended invasion to the intracranial and infratemporal fossa. No distant metastases of middle-ear carcinoid tumors have been reported. Therefore, middle-ear carcinoid tumors should be approached as low-grade malignant tumors [22], especially when assessing the cervical node status during the follow-up period. 

The 10-year survival rates for patients with carcinoid tumors and atypical carcinoid tumors among neuroendocrine tumors are 90% and 50%, respectively, whereas the survival rate is only 5% for patients with small-cell carcinomas. The distinction of a carcinoid tumor from small-cell carcinoma is critical because of major differences in the management and prognosis. There is a strong correlation between the proliferative index detected by Ki-67 labeling using MIB-1 and the grade of the tumor [23]. There is also a similar correlation between low Ki-67 values and longer survival in carcinoid tumors, but no clear cutoff value has been defined [23–26]. Şahan et al. reported that the Ki-67 labeling index was less than 1% in a patient with a middle-ear carcinoid [13]. As compared with the present case, the Ki-67 index was found to be 6.6% and appeared to show higher mitotic activity. The index may be a significant predictor for probability of osteolytic enlargement, local recurrence, and regional metastases of middle-ear carcinoids. 

## 4. Conclusions

The present report describes the case of a 59-year-old male patient who presented with a carcinoid tumor of the middle ear, and a partial petrosectomy was performed because the tumor had extended to the carotid artery canal and jugular bulb with bone erosion. A carcinoid tumor of the middle ear is not generally considered to represent a malignancy, but should be considered in the differential diagnosis of low-grade malignancies.
